# An innovative paradigm of intensive care: hot pot pattern

**DOI:** 10.3389/fmed.2025.1667837

**Published:** 2025-10-08

**Authors:** Kai Zhang, Yiping Zhou, Lanxin Cao, Yushi Fan, Wei Cui, Gensheng Zhang

**Affiliations:** ^1^Department of Critical Care Medicine, Zhejiang University School of Medicine Second Affiliated Hospital, Hangzhou, Zhejiang, China; ^2^Department of Critical Care Medicine, Zhejiang Cancer Hospital, Hangzhou, China; ^3^Key Laboratory of Multiple Organ Failure (Zhejiang University), Ministry of Education, Hangzhou, China

**Keywords:** intensive care, critically ill, paradigm, hot pot pattern, intensive care management

## Abstract

This paper proposes the novel “Hot Pot Pattern” to conceptualize intensive care. The conventional treatment and monitoring methods form the foundational “Hot Pot Base,” and individualized interventions form the “Hot Pot Ingredients.” This model elucidates the integration of standardized care with personalized medicine inherent in intensive care management.

## Introduction

The intensive care unit (ICU) is a critical component of modern hospitals, designed to provide comprehensive care for critically ill patients. Given the complexity and variable needs of critically ill patients, the methods of monitoring and treatment in the ICU must be both standardized and highly individualized (1). However, the current paradigm of intensive care faces significant challenges in balancing standardization with personalization. Medical students, residents, and even experienced clinicians often struggle to understand how to systematically approach the multifaceted nature of intensive care while simultaneously tailoring treatments to individual patient needs (2). There is a pressing need for an intuitive conceptual framework that can simplify the understanding of ICU care while maintaining its complexity and nuance.

To address this challenge, we propose an innovative analogy that conceptualizes the paradigm of intensive care as akin to a hot pot of Szechuan. This comparison serves several critical purposes: first, it provides an accessible mental model that can enhance comprehension of complex ICU protocols; second, it illustrates how standardized care (the base) can be seamlessly integrated with individualized treatments (the ingredients); and third, it offers a practical teaching tool that can improve medical education and clinical training in intensive care settings.

### What is hot pot?

Hot pot, also known as Chinese fondue, represents a distinctive form of Chinese cuisine. The concept revolves around a pot of boiling broth placed at the center of the table, which is also called the hot pot base; whereas a variety of individual ingredients are chosen by diners to create a rich and diverse dining experience. The success of hot pot lies in its perfect balance between a standardized foundation (the base) and unlimited customization possibilities (the ingredients). Similarly, the paradigm of intensive care can be viewed as a multi-layered and dynamically adjusted process like a hot pot.

### Conventional methods: the hot pot base

**Figure 1:**
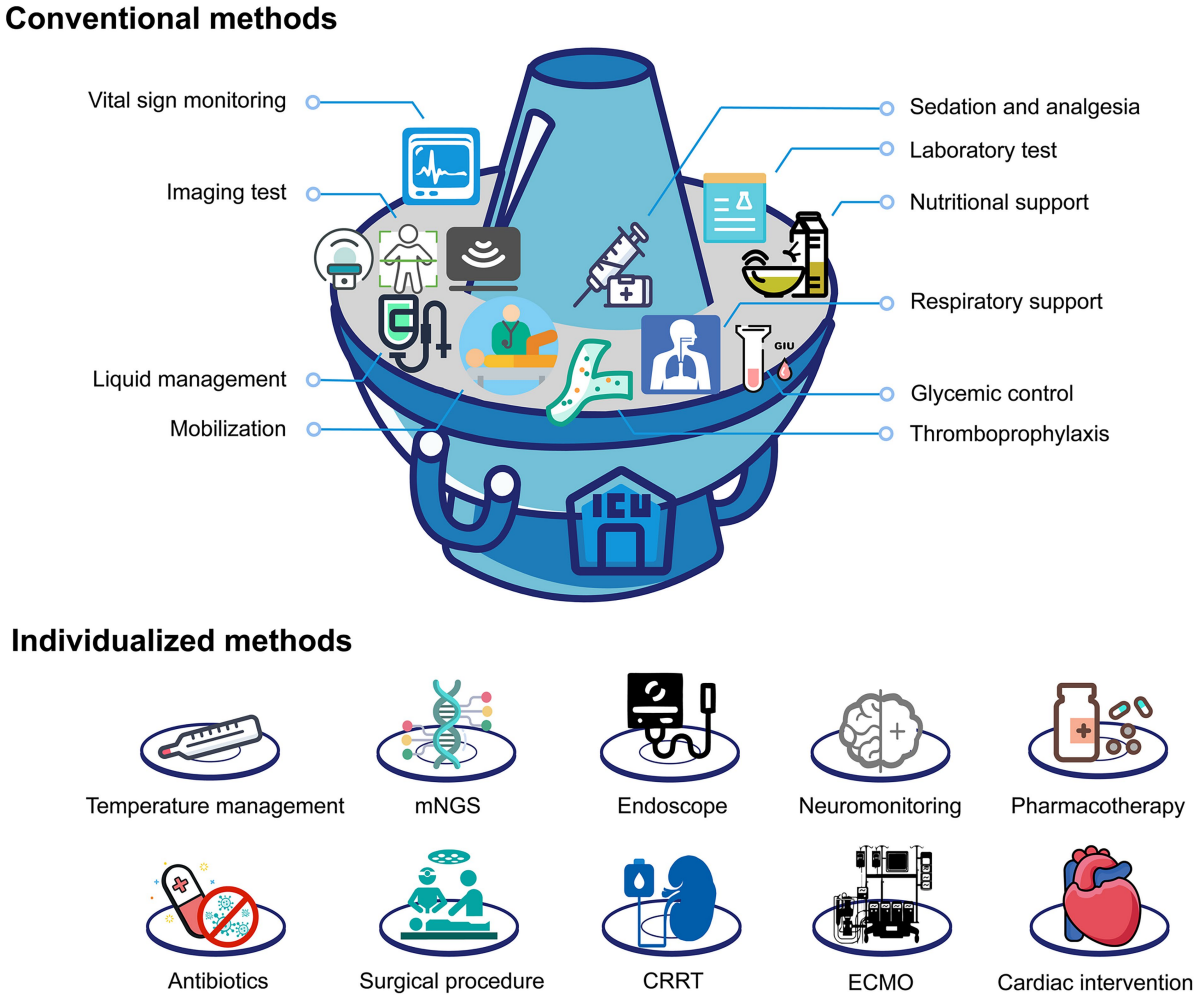
The paradigm of intensive care can be viewed as a hot pot.

In the ICU, conventional treatment and monitoring methods constitute the cornerstone of patient care, much like the base broth in a hot pot (Figure 1). These fundamental approaches include basic life support measures, routine vital sign monitoring, standard laboratory assessments, infection prevention protocols, and established clinical guidelines. Just as the hot pot base provides essential flavor and nourishment that forms the foundation for the entire meal, conventional treatment and monitoring are indispensable in the ICU, forming the backbone of intensive care. Without this solid foundation, no individualized intervention can be effective.

### Individualized methods: the hot pot ingredients

Various ingredients added to the hot pot provide unique flavors and nutritional value, each contributing to the overall dining experience based on individual preferences and dietary requirements. Similarly, individualized treatment and monitoring methods in the ICU are tailored to each patient’s specific conditions and needs. These may include specialized monitoring devices, targeted pharmacotherapy, organ-specific support systems, and personalized treatment protocols. Selecting and applying these individualized methods necessitate dynamic adjustments based on the patient’s changing condition, akin to selecting hot pot ingredients based on individual preferences and nutritional requirements (Figure 1).

### Example

**Figure 2:**
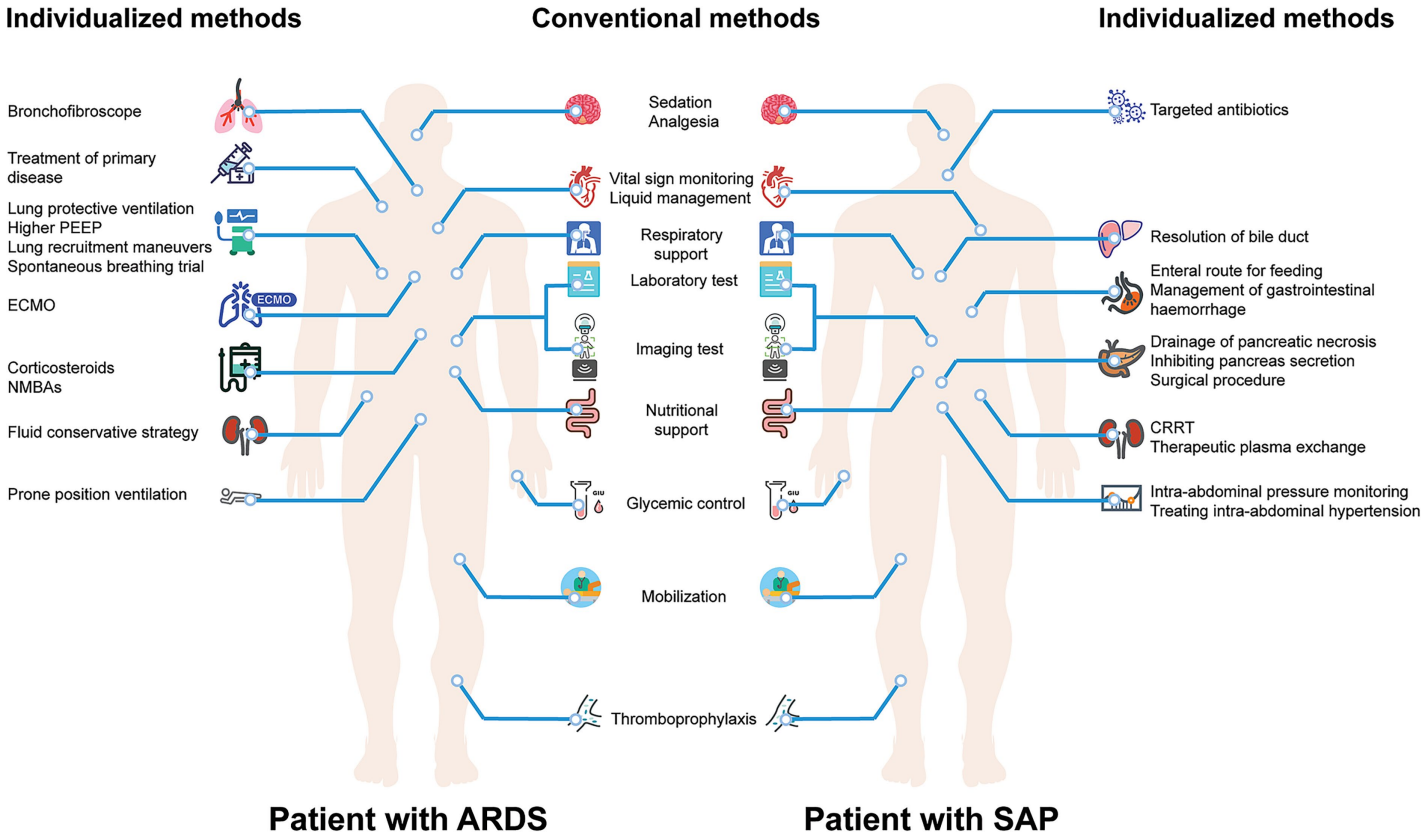
The application of hot pot pattern in critically ill patients with different diseases.

Figure 2 illustrates the application of the hot pot pattern in critically ill patients with different diseases. Upon admission of patients, whether with acute respiratory distress syndrome (ARDS) or severe acute pancreatitis (SAP) to the ICU, the initial step is similar to the hot pot base involving these conventional treatments and monitoring. For patients with ARDS, individualized treatments and monitoring including mechanical ventilation with specific parameters, treatment of primary disease, specific pharmacotherapy, specialized organ support, and conservative fluid strategy (3); while for patients with SAP, individualized treatment and monitoring including targeted antibiotics, drainage and surgical intervention, intra-abdominal pressure monitoring, plasma exchange, renal replacement therapy, and management of complications (4).

## Discussion

The paradigm of intensive care, conceptualized as a hot pot, highlights the complexity and flexibility inherent in intensive care medicine while providing a practical framework for clinical practice and medical education. This analogy addresses several critical aspects of modern intensive care that deserve detailed exploration.

### Enhanced clinical decision-making framework

The hot pot analogy provides clinicians with a systematic approach to intensive care that balances standardization with individualization. By establishing a clear distinction between the “base” (conventional care) and “ingredients” (individualized interventions), healthcare providers can ensure that fundamental care principles are never compromised while pursuing personalized treatment strategies. This framework reduces the cognitive burden on clinicians by providing a structured approach to complex decision-making processes.

### Educational advantages and training applications

This conceptual model offers significant advantages for medical education and training programs. The hot pot analogy creates an easily memorable and culturally relevant teaching tool that can bridge the gap between theoretical knowledge and practical application. Students and residents can rapidly grasp the concept that successful intensive care requires both a solid foundation of standard practices and the flexibility to adapt treatments to individual patient needs. This understanding can accelerate the learning curve and improve competency development in intensive care medicine (5).

### Interdisciplinary communication and team coordination

The hot pot model facilitates better communication among multidisciplinary ICU teams by providing a common conceptual framework. Nurses, physicians, respiratory therapists, pharmacists, and other healthcare professionals can more easily discuss and coordinate care plans when they share this unified understanding of how conventional and individualized care components integrate. This improved communication can lead to better team cohesion and more effective patient care delivery.

### Quality improvement and clinical outcomes

By ensuring that the “base” elements of care are consistently implemented while allowing for appropriate individualization, this paradigm can contribute to improved clinical outcomes and reduced practice variability. The framework encourages clinicians to maintain adherence to evidence-based standard protocols while providing clear guidance on when and how to implement personalized interventions. This balance is crucial for optimizing patient outcomes while maintaining cost-effectiveness.

### Future implications and precision medicine integration

As intensive care medicine continues to evolve toward precision medicine approaches, the hot pot paradigm provides a flexible framework that can accommodate emerging technologies and personalized treatment modalities. Advanced monitoring systems, artificial intelligence-guided interventions, and genomic-based therapies can all be conceptualized as sophisticated “ingredients” that complement the fundamental “base” of conventional care (6). This adaptability ensures that the model remains relevant as medical technology advances.

To maximize the benefits of this paradigm, healthcare organizations should consider systematic implementation strategies including: development of standardized “base” protocols that ensure fundamental care consistency (7), creation of decision-support tools that guide appropriate “ingredient” selection (8), integration of the model into medical education programs, and establishment of quality metrics that assess both standardization and individualization aspects of care.

With advancements in medical technology and intensive care research, the precision and efficacy of individualized ICU treatments will further improve (9). The hot pot paradigm provides a robust framework that can evolve with these advancements while maintaining its core principles. Furthermore, this analogy improves the comprehension and implementation of complex ICU treatments, which could be seen as a simple pattern of the combination of universal “Hot Pot Base” and individualized “Hot Pot Ingredients.” This pattern aids students and residents in rapidly understanding ICU treatment and monitoring strategies, creates an environment conducive to specialized training and interdisciplinary learning (10), ultimately leading to better healthcare for critically ill patients.

## Data Availability

The original contributions presented in the study are included in the article/supplementary material, further inquiries can be directed to the corresponding author.
